# Phase I study of photodynamic therapy using talaporfin sodium and diode laser for local failure after chemoradiotherapy for esophageal cancer

**DOI:** 10.1186/1748-717X-7-113

**Published:** 2012-07-23

**Authors:** Tomonori Yano, Manabu Muto, Kenichi Yoshimura, Miyuki Niimi, Yasumasa Ezoe, Yusuke Yoda, Yoshinobu Yamamoto, Hogara Nishisaki, Koji Higashino, Hiroyasu Iishi

**Affiliations:** 1 Department of Gastroenterology and Gastrointestinal Oncology, National Cancer Center Hospital East, Kashiwa, Japan; 2 Department of Gastroenterology and Hepatology, Kyoto University Graduate School of Medicine, Kyoto, Japan; 3 Translational Research Center, Kyoto University Hospital, Kyoto, Japan; 4 Department of multidisciplinary cancer treatment, Kyoto University Graduate School of Medicine, Kyoto, Japan; 5 Department of Gastroenterological oncology, Hyogo Cancer Center, Hyogo, Japan; 6 Department of Gastrointestinal Oncology, Osaka Medical Center for Cancer and Cardiovascular Diseases, Osaka, Japan; 7 Department of Gastroenterology and Hepatology, Kyoto University Graduate School of Medicine, 54 Kawahara-cho, Shogoin, Sakyo-ku, Kyoto, 606-8507, Japan

**Keywords:** Photodynamic therapy, Esophageal cancer, Talaporfin sodium, Salvage treatment, Phase I study

## Abstract

**Background:**

Photodynamic therapy (PDT) is a less invasive and effective salvage treatment for local failure after chemoradiotherapy (CRT) for esophageal cancer, however it causes a high rate of skin phototoxicity and requires a long sun shade period. Talaporfin sodium is a rapidly cleared photosensitizer that is expected to have less phototoxicity. This study was undertaken to clarify the optimum laser fluence rate of PDT using talaporfin sodium and a diode laser for patients with local failure after CRT or radiotherapy (RT) for esophageal cancer.

**Methods:**

This phase I, laser dose escalation study used a fixed dose (40 mg/m^2^) of intravenous talaporfin sodium administered 4 to 6 hours before irradiation in patients with local failure limited to T2 after CRT or RT (≥ 50 Gy). The primary endpoint was to assess the dose limiting toxicity (DLT) of PDT, and the secondary endpoints were to evaluate the adverse events and toxicity related to PDT. The starting fluence of the 664 nm diode laser was 50 J/cm^2^, with an escalation plan to 75 J/cm^2^ and 100 J/cm^2^.

**Results:**

9 patients with local failure after CRT or RT for ESCC were enrolled and treated in groups of 3 individuals to the third fluence level. No DLT was observed at any fluence level. Phototoxicity was not observed, but one subject had grade 1 fever, three had grade 1 esophageal pain, and 1 had grade 1 dysphagia. Five of 9 patients (55.6%) achieved a complete response after PDT.

**Conclusions:**

PDT using talaporfin sodium and a diode laser was safe for local failure after RT in patients with esophageal cancer. The recommended fluence for the following phase II study is 100 J/cm^2^.

## Background

Chemoradiotherapy (CRT) is one of the curative treatment options for esophageal cancer. However, local failure without distant metastasis after completion of CRT occurs in more than 40% of patients and this remains a major problem to achieve a cure [1]. Although salvage esophagectomy is generally indicated in such cases, it carries with it a high morbidity and mortality [2-5]. If the patients with local failure after completion of CRT have T2 or earlier T-stage residual tumors or for those without lymph node metastasis, salvage esophagectomy has curative potential [2-5]. Onozawa et al. reported that regional nodal failure within the field of elective lymph node irradiation was rare in patients achieving a complete response (CR) after CRT (1%; 95% confidence interval [CI], 0%–5%) for esophageal squamous cell carcinoma (ESCC) [6]. These data might indicate that if both lymph node and distant metastasis were controlled by CRT, local salvage treatment that targeted only the primary site could be a minimally invasive curative treatment option in carefully selected patients.

To develop such a treatment option for local failure, we have introduced photodynamic therapy (PDT) as a salvage treatment [7,8], and reported the results of the phase II study of salvage PDT for local residual T1 tumors after CRT [9]. In this study, 76% (19/25) of the patients could achieve CR and suffered only modest rates of adverse events and complications. These data suggest salvage PDT could be a curative treatment option for patients with local failure after CRT.

However, first generation PDT using porfimer sodium (Photofrin Injection, Pfizer Japan Inc., Tokyo, Japan) has some problems such as a high risk of skin phototoxicity requiring a long sun shade period (4–6 weeks), and the need for a large and expensive excimer dye laser system.

In contrast, second generation PDT using talaporfin sodium (Laserphyrin for Injection, Meiji Seika Pharma Co., Ltd., Tokyo, Japan) is featured as possessing a more rapid clearance from the skin and a longer absorption band (664 nm) compared with porfimer sodium. It is also theoretically expected to have a lower rate of phototoxicity with a shorter sun shade period and be more effective in deeper layers of tissue. Furthermore, the diode laser system (PD laser, Panasonic Healthcare Co., Ltd., Ehime, Japan) that emits 664 nm laser light and excites the talaporfin sodium is a much smaller system compared with the excimer dye laser system. In a clinical trial for early lung cancer treatment, PDT using talaporfin sodium demonstrated a high response rate (95%), similar to PDT using porfimer sodium, and modest skin photosensitivity with a 2 week sun shade period [10]**.** However, PDT using talaporfin sodium and diode laser is approved only for early lung cancer in Japan, and its safety and efficacy have not been clinically evaluated for other cancers.

The aim of this phase I study was to clarify the optimum laser irradiation fluence rate, and to evaluate the safety of PDT using talaporfin sodium and diode laser for patients with local failure after CRT or radiotherapy (RT) for esophageal cancer.

## Methods

This study was a multi-institutional open label phase I laser dose escalation study with a fixed dose of talaporfin sodium for patients with local failure after CRT or RT for ESCC. The study protocol was approved by the institutional review board of all participating institutions. This study was conducted in accordance with the Ethical Guideline for Clinical Research by the Ministry of Health, Labour and Welfare and the Declaration of Helsinki. The study was also registered with the University Hospital Medical Information Network (UMIN) Clinical Trials Registry, and the identification number is UMIN000003970.

### Patients

The eligibility criteria of this study were as follows: 1) local failure after CRT or RT(≥ 50 Gy) for esophageal cancer; 2) refusal of salvage esophagectomy or lack of tolerability for salvage esophagectomy; 3) local failed lesions limited within the muscularis propria (T2); 4) local failed lesions that were not involved in the cervical esophagus, 5) longitudinal lesion length of shorter than 3 cm and less than one half the circumference of the lumen; 6) no more than 2 failure lesions; 7) enrollments of patients with lymph node or distant metastasis were accepted, except for those with indication for systemic chemotherapy 8) local failure lesions which meet at least one of the following criteria; a) histologically proven carcinoma by biopsy specimen, b) emerged ulceration in the lesions, c) enlarged submucosal tumor like protrusion in the lesion, d) presence of heteroechoic solid component with endoscopic ultrasound (EUS) observation, 9) age ≥ 20, 10) Eastern Cooperative Oncology Group performance status ≤ 2; 11) adequate bone marrow function (white blood cell count ≥2000/mm^3^ and ≤ 12,000/mm^3^, hemoglobin >8.0 g/dL, platelet count ≥75,000/mm^3^), renal function (serum creatinine level ≤ 2.0 mg/dL), and liver function (serum total bilirubin level ≤ 2.0 mg/dL, both alanine aminotransferase and aspartate aminotransferase ≤ 100 IU/L); and 12) provision of written informed consent.

The exclusion criteria were 1) significant cardiovascular diseases (uncontrolled hypertension, myocardial infarction, unstable angina, congestive heart failure), uncontrolled diabetes mellitus, or severe liver cirrhosis; 2) systemic infection requiring antibiotics; 3) inability to obey the sun shade restrictions; 4) additional PDT just after salvage endoscopic mucosal resection or endoscopic submucosal dissection for local failures; 5) baseline lesions before CRT or RT judged to involve the aorta; 6) porphyria; 7) preexisting of sun photosensitivity; 8) previous treatment with PDT using talaporfin sodium, or treatment with PDT using porfimer sodium at least 3 months before enrollment; 9) pregnant or nursed women, or unwillingness to use of contraception; and 10) judged by investigator that enrollment was inappropriate for the patient.

### Study design

In the present study, the dose of talaporfin sodium was the same as that used for lung cancer, because the safety profile of the 40 mg/m^2^ dose was already clarified in the PDT regimen for lung cancer. Therefore, we planned this laser fluence escalation study to find the optimum fluence level of diode laser treatment for local failure after CRT or RT for esophageal cancer. The primary endpoint of this study was to assess the dose limiting toxicity (DLT) related with PDT at each level. The secondary endpoints were to evaluate the adverse events and toxicity related to PDT. The starting fluence of the diode laser was 50 J/cm^2^ (level 1), with the escalation plan increasing the fluence to 75 J/cm^2^ (level 2) and 100 J/cm^2^ (level 3). The starting fluence of 50 J/cm^2^ was chosen based on results of a pre-clinical study using canine esophagus model [11]. A minimum of 3 patients were assessed for toxicity at each level. If a DLT was not observed within 28 days after laser irradiation, then the level was raised. If a DLT was observed in one of the 3 patients, an additional 3 patients were treated at the same fluence level. The maximam tolerated dose (MTD) was defined at each level when DLT was observed in 2 or more of the 3 patients, or 3 or more of the two 3 patient groups that were treated at the same fluence level. The recommended dose (RD) for further study in phase II was defined as just below the level of the MTD. If the DLT was not observed in the level, then level 3 (100 J/cm^2^) was defined as the RD for the phase II study.

### Staging

In this study, clinical stage was determined according to the TNM classification of the International Union Against Cancer 6th edition [12] and the *Japanese Classification of Esophageal Cancer*, 10th edition, revised version [13]. Clinical T stage was evaluated by endoscopy, EUS, and computed tomography (CT) of the chest. Clinical N and M stages were evaluated by EUS and CT of the neck, chest, and abdomen.

### Treatment and surveillance

All PDT procedures were performed in an inpatient setting. One hundred milligrams of talaporfin sodium were dissolved in 4 mL of saline, and a 40 mg/m^2^ dose was slowly injected intravenously. Four to 6 hours after administration of talaporfin sodium, the local failure lesion was irradiated with diode laser at a 664 nm wavelength. The diode laser light was delivered via a straight type fiber without any balloon or light diffuser through the operative channel of the scope. A plastic attachment was fitted to the tip of the scope to keep it facing the lesion and to maintain the distance between the tip of the straight type fiber and the surface of the lesion during the procedure. If the lesions were larger than 1 cm^2^, multiple treatment fields were overlapped to cover the entire lesion. In this phase I study, the starting fluence was 50 J/cm^2^ (level 1), with a fixed fluence rate of 150 mW/cm^2^. If the post laser treatment change (e.g. ischemic change of mucosa) by endoscopic observation was suspected to be insufficient, additional laser irradiation was performed on the next day as a second session.

Before enrollment, patients were evaluated with a physical examination, performance status, dysphagia score [14] assessment, complete blood count, blood chemistry, electrocardiogram, and a chest-X ray study. After patient enrollment, adverse events were observed and graded until 28 days after laser irradiation. Patients were assessed through physical examinations, measurements of hematological and biochemical variables in the blood, chest X-ray studies, and endoscopic examinations, at least once a week until 28 days after PDT. All patients were instructed to avoid direct exposure to sunlight for 2 weeks after the injection of talaporfin sodium to protect them from skin photosensitization. Patients were allowed to discharge 2 weeks after laser irradiation if there were no complications related to PDT. Adverse events and toxicity were graded according to the Common Terminology Criteria for Adverse Events (CTCAE), version 4.0 [15].

DLT was defined as follows; 1) pain that requires administration of opioid analgesics for relief and persists for 4 days or more; 2) grade 2 or higher fever that persists 4 days or more, in spite of antipyretic administration; 3) grade 3 or higher esophageal fistula without any evidence of disease progression; 4) grade 3 or higher esophageal stenosis without any evidence of disease progression; 5) grade 3 or higher esophageal hemorrhage without any evidence of disease progression, 6) grade 4 or higher non-hematological toxicity.

### Efficacy

Treatment efficacy and toxicity at the primary site were evaluated by endoscopy every week for the first 4 weeks after PDT, and every 2 weeks thereafter until the efficacy was confirmed. The clinical criteria of CR at the primary site after PDT were as follows: 1) residual lesion not observed; 2) disappearance of post PDT ulceration and confirmation of the scar formation; and 3) histological confirmation of the absence of cancer cells by biopsy. In addition, when the ulceration or erosion was not cured within 6 months after PDT, but the biopsy specimen did not continuously reveal residual cancer cells, treatment efficacy was assessed as uncertain CR and they were evaluated as a CR case.

CT was used to evaluate distant organ or lymph node metastasis every 3 months after PDT. In this study, lymph node metastasis was diagnosed clinically if the lymph node was larger than 10 mm in diameter on CT, and distant metastasis was diagnosed if the emergent metastatic lesion was confirmed with CT.

## Results

### Patients characteristics

Between October 2010 to May 2011, a total of 9 patients with local failure after CRT or RT for ESCC were enrolled and treated in groups of 3 individuals using up to the third fluence level. Baseline patient and lesion characteristics before CRT or RT are shown in Table 1. All of the patients were male, and their median age was 72 years old (range: 58–83). The tumor location was the upper esophagus in one patient, middle esophagus in 5 patients, and lower esophagus in 3 patients. The baseline clinical stages before CRT or RT were: stage I in four, stage II in three, stage III in one, and stage IVb in one patient. The initial T stage was T1 in 4 patients, T2 in 2 patients, and T3 in 3 patients. Patient and lesion characteristics before PDT are shown in Table 2. Seven of the 9 patients were treated with CRT, and the other two were treated with RT alone. The irradiation dose for the patients was 60 Gy in 7 patients, 70 Gy in one patient, and 50.4 Gy in one patient, respectively. The majority of their chemotherapeutic regimen was cisplatin plus continuous infusion of fluorouracil. Their failure patterns were recurrence after achieving a CR with CRT in 7 patients, and residual tumors just after CRT in 2 patients. All local failure lesions enrolled in this study were histologically proven solitary lesion within the radiation field. The median tumor length was 1.0 cm (range: 0.8-3.0 cm), and the circumference of the lumen was 7 patients in < 1/4, and 2 patients in 1/4-1/2. The T stage of the recurrent lesion was T1 in 6 patients, and T2 in 3 patients. None of them had clinical lymph node or distant metastasis on CT evaluation before PDT.

**Table 1:** Baseline patient and lesion characteristics before RT

	**Age (y)**	**Gender**	**clinical stage**	**histology**	**Location**
Level 1 (50 J/cm^2^)					
No.1	74	Male	T3N1M1b	SCC	Middle
No.2	83	Male	T3N0M0	SCC	Middle
No.3	72	Male	T2N0M0	SCC	Middle
Level 2 (75 J/cm^2^)					
No.4	58	Male	T3N1M0	SCC	Middle
No.5	81	Male	T1N0M0	SCC	Lower
No.6	67	Male	T1N0M0	SCC	Middle
Level 3 (100 J/cm^2^)					
No.7	77	Male	T1N0M0	SCC	Upper
No.8	66	Male	T2N1M0	SCC	Lower
No.9	65	Male	T1N0M0	SCC	Lower

**Table 2:** Patient and lesion characteristics before PDT

	**radiation dose (Gy)**	**concomitant chemotherapeutic regimen**	**pattern of failure**	**number of lesion**	**histological confirmation**	**tumor length (cm)**	**lesion circumference of the lumen**	**clinical stage**
Level 1 (50 J/cm^2^)								
No.1	60	cisplatin + 5FU	residue	1	positive	2.0	<1/4	T2N0M0
No.2	60	nedaplatin + 5FU	recurrence	1	positive	1.0	<1/4	T1N0M0
No.3	60	5FU	residue	1	negative	1.5	<1/4	T2N0M0
Level 2 (75 J/cm^2^)								
No.4	60	cisplatin + 5FU	recurrence	1	positive	1.0	<1/4	T1N0M0
No.5	60	-	recurrence	1	positive	0.8	<1/4	T1N0M0
No.6	60	-	recurrence	1	positive	2.0	1/4-1/2	T1N0M0
Level 3 (100 J/cm^2^)								
No.7	60	cisplatin + 5FU	recurrence	1	positive	1.0	1/4-1/2	T1N0M0
No.8	50.4	cisplatin + 5FU	recurrence	1	positive	3.0	<1/4	T2N0M0
No.9	70	cisplatin + 5FU	recurrence	1	positive	1.0	<1/4	T1N0M0

### Toxicity

All 9 patients received administration of talaporfin sodium and diode laser irradiation, therefore they were assessable for DLT. No dermatological adverse events related to talaporfin sodium, such as allergic reaction or photosensitivity, were not observed in any patients. DLT was not observed in any patients at any level of treatments. The hematological and non-hematological toxicities in this study are summarized in Table 3. The hematological toxicity related to PDT was not observed in all patients, and when present, the non-hematological toxicity was generally mild and graded 1 as follows; 1 patient (11%) with grade 1 fever, 3 patients (33%) with grade 1 esophageal pain, and 1 patient (11%) with grade 1 dysphagia.

**Table 3:** Hematological and non-hematological toxicity

	**Level 1(50 J/cm**^**2**^**), n = 3**				**Level 2(75 J/cm**^**2**^**), n = 3**				**Level 3(100 J/cm**^**2**^**), n = 3**				**Total (any grade, %)**
	**Grade**				**Grade**				**Grade**				
	1	2	3	4	1	2	3	4	1	2	3	4	
Anemia	0	0	0	0	0	0	0	0	0	0	0	0	0
White bloode cell decreased	0	0	0	0	0	0	0	0	0	0	0	0	0
Neutrophil count decreased	0	0	0	0	0	0	0	0	0	0	0	0	0
Platelet count decreased	0	0	0	0	0	0	0	0	0	0	0	0	0
Fever	0	0	0	0	1	0	0	0	0	0	0	0	1(11%)
Esophageal pain	1	0	0	0	2	0	0	0	0	0	0	0	3(33%)
Pharyngolaryngeal pain	0	0	0	0	0	0	0	0	0	0	0	0	0
Dysphagia	1	0	0	0	0	0	0	0	0	0	0	0	1(11%)
Anorexia	0	0	0	0	0	0	0	0	0	0	0	0	0
Nausea	0	0	0	0	0	0	0	0	0	0	0	0	0
Photosensitivity	0	0	0	0	0	0	0	0	0	0	0	0	0

### Efficacy

The details of the procedure and treatment efficacy of PDT are summarized in Table 4. The esophageal surface areas that were treated ranged 2–3 cm^2^ in level 1, 2–4 cm^2^ in level 2, 1–2 cm^2^ in level 3, respectively. The total irradiation dose ranged 100–150 J in level 1, 150–300 J in level 2, and 100–200 J in level 3, respectively. A CR was achieved in 5 of 9 patients (55.6%, 95% Confidence interval [CI]: 21.2–86.3). The CR rate of each fluence level was 33.3% (1/3) in level 1, 66.7% (2/3) in level 2, and 66.7% (2/3) in level 3, respectively. Total 2 of 9 patients irradiated with another session on the next day. One was at the level 1 (patient No.3), and the other was at level 3 (patient No.8). Patient No.3 could be achieved CR, whereas No.8 could not be. There was no severe toxicity with additional irradiation on the next day.

**Table 4:** The details of procedure and the efficacy of PDT

	**treated surface area (cm2)**	**total irradiation dose (J)**	**response of PDT**	**the period between PDT and CR (days)**	**local recurrence after CR**	**metastasis after CR**
Level 1 (50 J/cm^2^)						
No.1	3	150	nonCR	-	-	-
No.2	2	100	nonCR	-	-	-
No.3	3	150	CR	99	No	No
Level 2 (75 J/cm^2^)						
No.4	2	150	nonCR	-	-	-
No.5	2	150	CR	63	No	Yes (LN)
No.6	4	300	CR	29	No	No
Level 3 (100 J/cm^2^)						
No.7	1	100	CR	39	Yes	No
No.8	2	200	nonCR	-	-	-
No.9	1	100	CR	33	Yes	No

CR complete response, LN lymph node.

**Figure 1:**
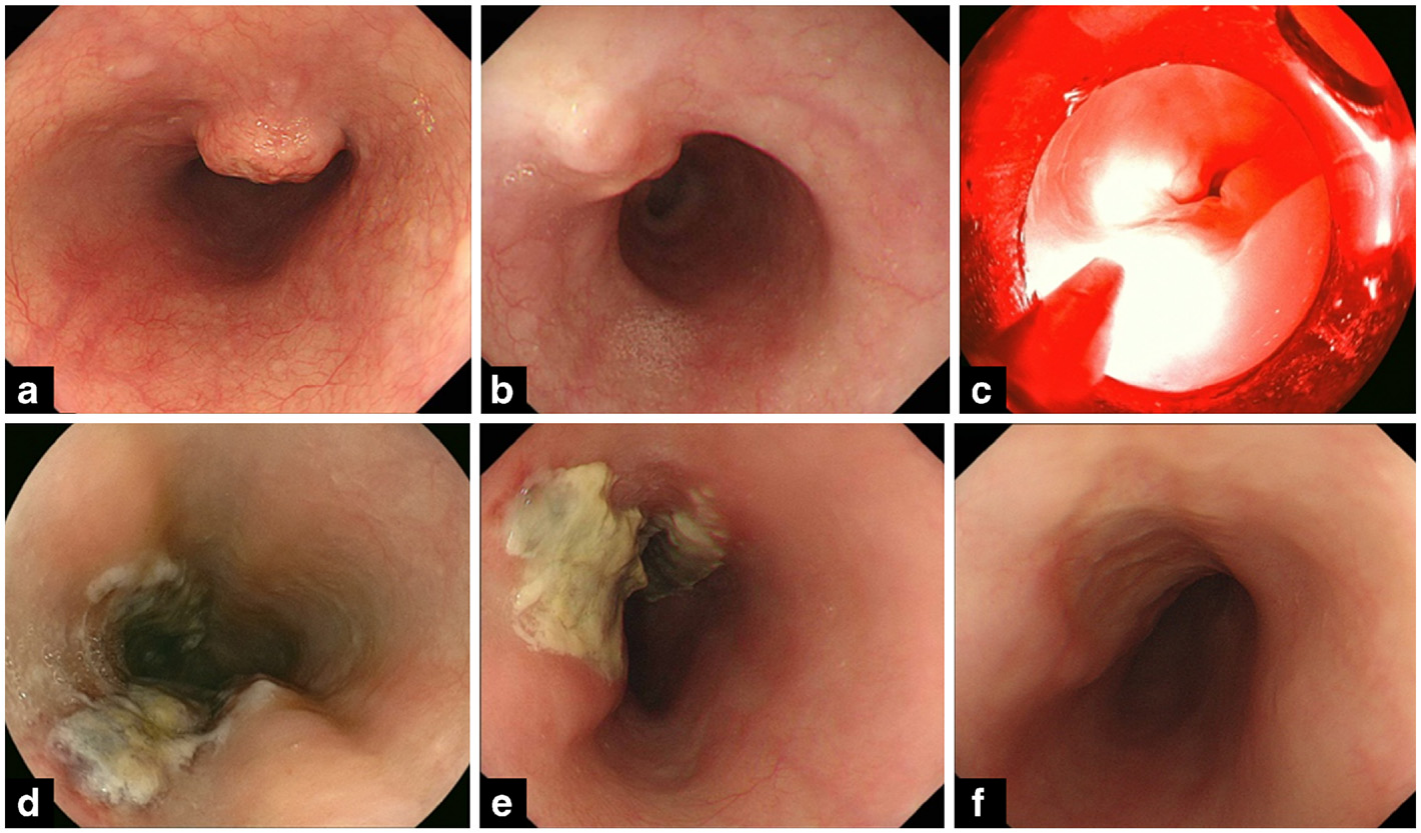
**A patient who achieved complete response with photodynamic therapy**. **a**: before chemoradiotherapy. **b**: local residue was detected after chemoradiotherapy. **c**: during PD laser irradiation. **d**: two days after PDT, ischemic change was observed at the laser irradiated site. **e**: two weeks after PDT, deep ulceration was observed at the laser irradiated site. **f**: CR was achieved 10 weeks after PD.

The median period between PDT and the confirmation of CR at primary site was 39 days (range: 29–99). A representative case of a patient who achieved CR is shown in Figure 1**.** Furthermore, the CR rate of patients with T1 local failure was 66.7% (4/6), and that of patients with T2 local failure was33.3% (1/3). At the median follow up period of 275 days (range: 91–371), of the 5 patients who achieved CR with PDT, local recurrence was detected in 2, and the lymph node metastasis was detected in one patient.

## Discussion

In this study, we evaluated for the first time the efficacy and safety of PDT using talaporfin sodium and diode laser for patients with ESCC as well as for salvage treatment for the local failure after CRT or RT. We did not experience any DLT at any levels of laser irradiation and also had a promising CR rate, therefore PDT using talaporfin sodium and diode laser for esophageal cancer could be a tolerable therapeutic option.

In the present study, skin photosensitivity did not develop in any patients with 2 weeks of sun shade period. As for other adverse events, we experienced one patient (11%) in fever, 3 (33%) in esophageal pain, and 1 (11%) in dysphagia. However, these toxicities were grade 1. In contrast, in a prior phase II study of PDT using porfimer sodium and excimer dye laser, approximately 32% of the patients experienced photosensitivity in spite of 4 to 6 week sun shade period. Furthermore, we observed esophageal pain (61%), pharyngolaryngeal pain (17%), dysphagia (39%), and fever (48%) [9]. These toxicities were grade 1 or 2. These results indicated that talaporfin -based PDT had merits of less skin photosensitivity and a shorter sun shade period. However, because the esophageal surface area irradiated (1–4 cm^2^ vs 3–9 cm^2^) and the total laser irradiation dose (100–300 J vs 225–675 J) were quite different between each study, the toxicity related with laser irradiation should be evaluated in the next phase II study.

While this study was a phase I study, 5 of 9 patients (55.6%) achieved a CR. The CR rate for the T1 residual tumors was 66.7%, and that of T2 was 33.3%. These data were similar to the efficacy with PDT using porfimer sodium with excimer dye laser. This indicated that PDT had a high potential for local control for ESCC. Although further evaluation for the efficacy of talaporfin-based PDT is necessary in the next step with larger subjects, talaporfin-based PDT demonstrated promising anti-tumor effect for the ESCC.

Second-line systemic chemotherapy is also a treatment option for patients with treatment failure after CRT for ESCC. However, systemic chemotherapy has not demonstrated a sufficient effect in studies; the overall response rate ranged 0%–16%, and the complete response rate ranged 0%–6% [16-19]. Even for the primary site, it is quite difficult to achieve CR. This suggests that second-line systemic chemotherapy is most likely a palliative treatment option. Taken together, salvage PDT could be a curative treatment option for local failure after CRT.

For treatment failure after CRT for ESCC, salvage surgery is generally indicated. However, it demonstrated a high rate of complications and treatment-related mortality ranging from 8% to 22% [2-5]. Therefore, the indications for salvage surgery should be carefully considered. In this study, there were no severe adverse events related to talaporfin sodium-based PDT. Although there was one case of treatment related death in the prior phase II study of PDT using porfimer sodium, the rate of treatment related death was only 4% (1/25). We believe, based on these results, that salvage PDT is a less life-threating treatment option than salvage surgery.

Before starting this study, we performed a pre-clinical study of PDT using talaporfin sodium and diode laser for canine esophagus [11]. In that pre-clinical study, laser irradiation was escalated with three levels of fluence 25, 50, 100 J/cm^2^ after administration of talaporfin sodium for three dogs at each levels, and pathologically evaluated one week after irradiation. The ulceration and ischemic changes around the ulcer became more severe as the dose of laser irradiation increased. Pathologically, these changes were confined to the mucosa after irradiation at 25 J/cm^2^, whereas they appeared as necrosis in the muscle layer after irradiation at 50 J/cm^2^, and the necrosis extended to the extra-adventitial tissue after irradiation at 100 J/cm^2^. Therefore, we concluded that 25 J/cm^2^ was within the safe range in normal canine esophagus. However, this dosage cannot directly apply for human, because the thickness is different between human and dog esophagus. Previous data on the normal canine bronchi and a clinical study of human lung cancer suggest that similar effects should be expected in humans after double-irradiation doses that were used in dogs. Therefore, we recommend that human clinical trials in the esophagus should start with an irradiation dose of 50 J/cm^2^, and it might be appropriated from the safety profile of present study. In conclusion, PDT using talaporfin sodium and diode laser irradiation demonstrated an acceptable safety profile with manageable adverse events and promising efficacy for local failure after RT for patients with esophageal cancer. From the results of this study, 100 J/cm^2^ was selected as a recommended fluence for the next phase II study.

## Abbreviations

CRT, Chemoradiotherapy; CR, Complete response; ESCC, Esophageal squamous cell carcinoma; PDT, Photodynamic therapy; RT, Radiotherapy; UMIN, University hospital Medical Information Network; EUS, Endoscopic ultrasound; DLT, Dose limiting toxicity; MTD, Maximam tolerated dose; CTCAE, Common terminology criteria for adverse events.

## Competing interests

There are no financial disclosure or conflict of interest relevant to this study.

## Authors’ contributions

TY, MM, YE,YYO, YYA, HN, KH, HI are responsible for patient accrural, treatment and follow up. TY, MM, KY, MN are responsible for study design, protocol and draft of the manuscript. KY is a statisticain and MN is a cordinator of this study. MM organised this group and study. All authors read and appoved the final manuscript.

## Financial support

This study was supported by Research grant of Ministry of Health, Labour and Welfare of Japan.
